# In-hospital resuscitation evaluated by in situ simulation: a prospective simulation study

**DOI:** 10.1186/1757-7241-19-55

**Published:** 2011-10-06

**Authors:** Frederik Mondrup, Mikkel Brabrand, Lars Folkestad, Jakob Oxlund, Karsten R Wiborg, Niels P Sand, Torben Knudsen

**Affiliations:** 1 Sydvestjysk Sygehus Esbjerg, Department of Emergency Medicine, Finsensgade 35, DK-6700 Esbjerg, Denmark; 2 Sydvestjysk Sygehus Esbjerg, Department of Anaesthesiology, Finsensgade 35, DK-6700 Esbjerg, Denmark; 3 Sydvestjysk Sygehus Esbjerg, Department of Cardiology, Finsensgade 35, DK-6700 Esbjerg, Denmark; 4 Institute of Regional Health Services Research, University of Southern Denmark, Denmark; 5 Sydvestjysk Sygehus Esbjerg, Department of Medical gastroenterology, Finsensgade 35, DK-6700 Esbjerg, Denmark

**Keywords:** cardiopulmonary resuscitation, simulation, in-situ simulation, no flow ratio, no flow time

## Abstract

**Background:**

Interruption in chest compressions during cardiopulmonary resuscitation can be characterized as no flow ratio (NFR) and the importance of minimizing these pauses in chest compression has been highlighted recently. Further, documentation of resuscitation performance has been reported to be insufficient and there is a lack of identification of important issues where future efforts might be beneficial. By implementing in situ simulation we created a model to evaluate resuscitation performance. The aims of the study were to evaluate the feasibility of the applied method, and to examine differences in the resuscitation performance between the first responders and the cardiac arrest team.

**Methods:**

A prospective observational study of 16 unannounced simulated cardiopulmonary arrest scenarios was conducted. The participants of the study involved all health care personel on duty who responded to a cardiac arrest. We measured NFR and time to detection of initial rhythm on defibrillator and performed a comparison between the first responders and the cardiac arrest team.

**Results:**

Data from 13 out of 16 simulations was used to evaluate the ability of generating resuscitation performance data in simulated cardiac arrest. The defibrillator arrived after median 214 seconds (180-254) and detected initial rhythm after median 311 seconds (283-349). A significant difference in no flow ratio (NFR) was observed between the first responders, median NFR 38% (32-46), and the resuscitation teams, median NFR 25% (19-29), p < 0.001. The difference was significant even after adjusting for pulse and rhythm check and shock delivery.

**Conclusion:**

The main finding of this study was a significant difference between the first responders and the cardiac arrest team with the latter performing more adequate cardiopulmonary resuscitation with regards to NFR. Future research should focus on the educational potential for in-situ simulation in terms of improving skills of hospital staff and patient outcome.

## Introduction

Recent investigations highlight the importance of reducing interruptions in chest compressions and early defibrillation as vital factors of cardiopulmonary resuscitation (CPR), and the European Resuscitation Council 2010 Guidelines (ERC 2010) further emphasize these elements [1-9].

Despite clear recommendations on CPR performance, several studies reports insufficient CPR quality during training (simulation) and during out-of-hospital and in-hospital cardiac arrests [10-14]. Documentation of resuscitation management may be difficult in the acute situation and it has been reported to be insufficient [15,16]. Furthermore, the retrospective nature of documentation in records represents a pitfall due to incompletion or inaccuracy [17]. Documentation regarding precise timing of events during resusciation, such as data concerning chest compressions and defibrillation, represents a problem and data may be imprecise or not even available [16]. Thus, a gap exists in documentation between first responders and cardiac arrest team, and the inadequate documentation may lead to misinterpretation in resuscitation performance. Finally, data from patient safety incidents and adverse event reporting systems suffers from underreporting [18]. Due to these problems, there is a lack of identification of issues which need attention and where future efforts might turn out to be beneficial.

Medical simulation has become widespread and plays a central role in teaching and in the assessment of doctors and other health care professionals [19].

Simulation performed within a clinical enviroment, *in situ simulation*, is particularly suitable to identify system weaknesses or errors and to perform context-sensitive assessments. By bringing simulation into the clinical enviroment, it is possible to identify and prevent adverse events that could compromise patient safety [20-22]. Furthermore, in situ simulation represents a cost-effective opportunity in medical education and several studies report the utility of simulation training for acquisition of skills and knowledge with retention across different specialities [23-25].

By implementing in situ simulation to perform unannounced in-hospital cardiopulmonary arrest, we created a model to evaluate resuscitation performance.

Our aims in this study were to: 1) evaluate the feasibility of the applied method and ability to generate resuscitation performance data, 2) examine whether there is a difference in the resuscitation performance between first responders and the cardiac arrest team in unannounced simulated scenarios.

## Methods

### Design

The study was a prospective simulation pilot study which evaluated the resuscitation performance during simulated cardiac arrest. The local ethical committee was queried and the decision "ethical approval not required" was given. Danish law exempts this type of research from ethical approval. The board of the hospital and all involved heads of departments were informed about the purpose of the study and gave their consent to participate.

### Setting and participants

The study was conducted in a regional teaching hospital with approximately 500 beds and an annual census of approximately 43,000 patients.

Data collection consisted of data registered during unannounced simulated cardiac arrests in the period April 2010 - June 2010. The simulations were conducted in day-time only.

The participants of the study involved all health care personel on duty who are expected to respond to a cardiac arrest. This involves first responders, typically nurses or nurse-assistants who identify the cardiac arrest, call the cardiac arrest team and initiate basic CPR. The cardiac arrest team assembles ad hoc and consists of a medical resident who serves as a team leader accompanied by a medical intern, an anesthesia resident and nurse, and two orderlies. The team is characterized by a wide disparity in clinical experience. The role of the orderlies is to secure arrival of the defibrillator, emergency equipment and to perform chest compressions.

### Scenario Setting

The study group developed four different on-site simulated scenarios with a resuscitation manikin Resusci Anne Simulator (Laerdal Medical ^®^, Stavanger, Norway) for interdisciplinary resuscitation. The Lifepak 12 (Medtronic^®^, Redmond, United States of America) defibrillator was used throughout the study and only in manual mode. The scenarios were conducted in two units of the hospital (a surgical and a medical unit) and featured common causes to cardiac arrest e.g. chest pain, hypoxia and hypovolemia.

Furthermore, each scenario had pre-defined scripted branch-points from start to stop and included both shockable and non-shockable rhythms. The scenarios would advance according to actions of the first responders and the cardiac arrest team. Finally, each scenario had a patient background file with a brief medical history and test results to provide additional immersion.

### Sequence of events

The nurse manager of the ward was contacted in advance, and a room and a covering nurse were assigned. All equipment including manikin, laptop, three remote controlled moveable cameras, and a microphone were quickly installed by a technician. The nurse assigned to the room was introduced to the simulated patient and the medical history, and was instructed to intervene as one would do with a regular patient. The scenario developed into cardiac arrest and the additional personnel assembling to the simulation were unaware of the ongoing mock event. They were instructed to respond according to their clinical responsibilities upon arrival at the patient, e.g. the first responders initiated basic resuscitation. The scenario was ongoing and the first responders were released by the resuscitation team as they arrived. The assessment was performed in the two groups during the entire scenario. At the end of the simulation, two members of the study group performed a debriefing of the resuscitation. Investigators monitored other emergencies to prevent conflict with real emergencies and in case of an acute situation during simulation, this would lead to immediate interruption of the scenario and data was discarded. The investigators' roles were only observational and they would only interact with the personnel in order to prevent hazardous situations e.g. unsafe defibrillation and help to apply the modified defibrillation pads.

### Data collection and processing

All performance data were collected with Laerdal PC SkillReporting System version 2.0 (Laerdal Medical, Stavanger, Norway).

We defined no flow time (NFT) as the time from the onset of cardiac arrest (Time 0) to ROSC in which no chest compressions were being performed. Furthermore, we defined the no flow ratio (NFR) as the ratio between NFT and the total time of cardiac arrest (Time 0 to ROSC) [26]. This represents the fraction of time during resuscitation in which the circulation is compromised.

According to the ERC 2005 Advanced Life Support (ALS) Guidelines interval between stopping compressions and delivering a shock must be minimized [27]. We adjusted (NFT_adj_) for the time required for these procedures and a maximum of 5 seconds was given to rhythm analysis and 10 seconds to charge the defibrillator and shock delivery (when appropriate) per two minutes cycle. Ten seconds were allowed for pulse checks every two minutes. Hereby the NFT_adj _represents the potential for reducing time without circulation and would ideally be zero according to ERC 2005 Guidelines [13]. In addition we used the NFT_adj _to calculate the NFR_adj_, which represents the fraction of time during resuscitation with compromised circulation excluding time to the abovementioned obligate maneuvers.

Each resuscitation scenario was divided into 30-second segments, and NFT were measured. By using cameras we were able to identify the exact change in time of first responders and the cardiac arrest team as well as determination of return of spontaneous circulation (ROSC). All personnel were identified and registered with unique identification numbers to subsequently monitor any repeated participation and to remain subject anonymity and confidentiality.

Finally, we determined the time from recognition of cardiac arrest to initiation of CPR, the time to arrival of the defibrillator in the room, and the time to the first rhythm on the defibrillator. Time span for the first responders was defined as recognition of cardiac arrest (time 0) to arrival of one physician and orderlies. The resuscitation team time span was defined from end of first responders to completion of the scenario. Time to first rhythm on the defibrillator was defined as recognition of the cardiac arrest (time 0) to the first rhythm on the defibrillator's scope.

### Data analysis

All processed data from simulations was gathered using a spreadsheet application, Excel 2003 (Microsoft Corp.). All statistical analyses were performed with SPSS 15.0 (SPSS Inc, Chicago). As data was not normally distributed, data is presented as medians and interquartile ranges (25%- 75% percentile). We assessed differences in NFR using a nonparametric Mann-Whitney test. P-values below 0.05 were considered statistically significant.

## Results

We conducted 16 simulations and data from 13 was collected since two simulations were excluded due to other emergencies, and one simulation due to failure of transferring data.

There was no repeated participation among the first responders or assisting nurses during the simulations. One of the orderlies was involved in three different simulations. The participation registration showed that one physician was involved in three simulations and two different physicians participated in two simulations each (data not shown).

Overall, cardiopulmonary resuscitation performance data from simulated scenarios are summarized in table 1. During simulations we recorded a median compression rate of 117 compression min^-1 ^(112-122) and the actual delivered compression min^-1 ^were 82 (78-87). We observed that initiating of CPR during simulation was performed with a median of 29 seconds (22-46). The defibrillator arrived in the room after median 214 seconds (180-254) and it was used to detect initial rhythm after median 311 seconds (283-349).

**Table 1:** Cardiopulmonary resuscitation quality markers obtained from unannounced simulated cardiac arrest (n = 13)

	Overall simulation		
		Percentiles	
	Median	25	75
Compression rate (comp/min)	117	112	122
Compressions (actual comp. given/min)	82	78	87
Time to initiating of CPR (sec)	29	22	46
Time to arrival of defibrillator in room (sec)	214	180	254
Time to first rhythm on defibrillator (sec)	311	283	349
NFR (%)	28	23	31
NFR_adj (%)	18	13	22

CPR: Cardiopulmonary Resucitation

NFR: no flow ratio; percentage of the time during resuscitation without chest compressions and spontaneous circulation.

The median NFR for the entire simulation was 28% (23-31) and the adjusted median NFR (NFR_adj_) was 18% (13-22).

Comparison of NFR between the first responders and the resuscitation teams are summarized in table 2. NFR for the first responders was median 39% (32-46) versus a median NFR 25% (19-29) for the cardiac arrest teams, p < 0.001. NFR_adj _for the first responders was a median 26% (22-38) versus NFR_adj _of 13% (11-17) for the resuscitation teams, p < 0.001. We performed a revised analysis without the simulations, which included repeated presence of the same physician, and the results did not change significantly (data not shown).

**Table 2:** Comparison of no flow ratio (NFR) between first responders and resuscitation team during unaccounced simulated cardiac arrest (n = 13)

	**First responders**			**Resuscitation team**			
		**Percentiles**			**Percentiles**		
	**Median**	**25**	**75**	**Median**	**25**	**75**	**p-value**
	
NFR (%)	39	32	46	25	19	29	p < 0.001†
NFR_adj (%)	26	22	38	13	11	17	p < 0.001†

NFR: no flow ratio; percentage of the of the time during resuscitation without chest compressions and spontaneous circulation.

NFRadj: no flow ratio_adjusted; percentage of the of the time during resuscitation without chest compressions and spontaneous circulation adjusted by subtraction of time allowed for rhythm and pulse check and defibrillation (when appropriate).

† Mann-Whitney test

## Discussion

There is, to our knowledge, no existing validated tool to identify errors and accurate assessment of NFR during CPR, due to a combination of practical restraints, research and simulation limitations. We achieved high realism during the simulations in a clinically familiar environment and thereby created an almost replication of a true cardiac arrest incident. Thus, we could generate data on simulated cardiac arrest response and performance and made it possible to assess the quality during the existing gap between first responders and cardiac arrest team. By using in situ simulation, we were able to establish a feasible model for studying unannounced simulated cardiac arrest scenarios in the systematic evaluation of cardiopulmonary resuscitation performance. We were able to objectively assess performance with regards to initiation of CPR, NFR and defibrillation during simulation.

The main finding of this study was a significant difference between the first responders and the cardiac arrest team with the latter performing more adequate cardiopulmonary resuscitation with regard to NFR. Other interview- and survey-studies present similar findings [28,29]. The results highlight the importance of the first initiated response as previously reported [20]. We monitored possible repeated staff participation and found no individuals performing multiple simulations as first responders. In the resuscitation team there were two individuals (one of the orderlies and one physician) who attended three simulations each. We do not believe that this observation may explain the significant difference but it may tend to favour the no flow ratio in the resuscitation group due to familiarity of the study setup. We performed a revised analysis excluding these simulations and the results did not change significantly. The main reason for the difference in performance was not systematically analysed but we observed a tendency in delaying initial CPR due to the performance of other tasks (data not shown). The difference could be due to lack of training and education, and this study might help clarify the first responders' task and importance in future education and training of cardiac arrest.

Surprisingly, we observed that the median time for arrival of the defibrillator was more than three and a half minutes which does not meet the current recommendations of two minutes [9]. This could be due to the fact that the orderlies only have access to one central defibrillator instead of multiple defibrillators in carefully selected locations in our hospital. Furthermore, it took more than five minutes to deliver a connected and powered defibrillator. An explanation could be unfamiliarity with the defibrillator despite training of the physicians. We observed several problems with finding and applying cables and pads (data not shown). There is also a risk that this could be due to the application of the modified study-pads.

## Limitations

There are several limitations in this study. First, the study is an analysis of simulated resuscitations and we are aware that the simulations may not represent actual responses during real cardiac arrests. As mentioned in the introduction, there is growing evidence that simulation can be used as a skills assessment tool. By using standardized pre-scripted scenarios, we attempted to minimize the gap in translating results from simulation to real life events. We did not correlate data recorded from real cardiac arrest to assure concordance due to the numbers of simulations. However, we observe data that seems comparable with data from previously published studies [13,14].

Secondly, participants may not have been fully immersed in the simulations due to e.g. personal reasons. This could bias the data towards giving performance of inferior quality and by artificially prolonging the initiation of CPR and time to first rhythm on defibrillator due to unfamiliarity with the simulation (equipment and environment). There is also a risk that the staff performed better due to the Hawthorne effect [30].

Thirdly, we only performed thirteen simulations which raise the possibility of producing non-representative data. Some of the hospital staff may never have attended the simulations and others several times due to staffing assignments.

Finally, we only conducted simulations on weekdays during daytime which might represent a bias in the resuscitation performance due to better staffing and better-performing staff. This applies to both first responders and the cardiac arrest team.

## Institutional impact

This study describes a model to monitor the quality of cardiopulmonary resuscitation as well as a tool to identify and prevent adverse incidents that could compromise patient safety. By conducting multiple simulations, we were able to generate objective resuscitation performance data and were able to monitor and document the quality of cardiac resuscitation and identify areas in need of improvement and also detect problems which would not have been found in other ways. We generated objective performance data during simulation and were able to identify prolonged defibrillation times concerning the physicians' handling of the defibrillator. We observed an in-adequate first response and while debriefing the exercises, we emphasized the importance of chest compressions and rapid defibrillation. Furthermore, these observations lead to a clarification of ward staff instructions in order to perform and act as first responders, and the experience was passed to the local educational panel.

Finally, our discoveries of prolonged defibrillation lead to a local discussion of introducing automated external defibrillators or multiple defibrillators.

## Perspectives

Future studies involving in situ simulation should evaluate the educational interventions' impact on performance and whether it can be used to improve clinical performance and hopefully improve patient outcome. Furthermore, educational studies should evaluate parameters such as leadership, communication and teamwork with standardised assessment tools such as Cardioteam as these are calibrated and validated for in situ simulation [31].

Finally, application of in situ simulation can provide considerable information with identification of system-level problems and performance assessment not only in cardiac arrest resuscitation but also in other areas of medical and surgical therapies. Furthermore, in situ simulation is suitable for identifying logistical and operational problems in institutions as the ongoing merging of emergency departments and new extensions occur.

In situ simulation can be used alone to generate more precise data on timing of events together with information concerning leadership, human factors and operational and low-practical bed-side findings. In combination with chart reviews and patient safety incident reports, these modalities can serve each other as complementary and reduce the risk of misjudgment system performance and lack of recognition of shortcomings as previously proposed [17].

## Conclusion

In situ simulation provides a safe opportunity to investigate performance on an organizational as well as bed-side level. We applied in situ simulation and were able to assess cardiopulmonary resuscitation without compromising patient safety, and we believe that in situ simulation could be used as a supplementary tool to assess cardiopulmonary resuscitation. We observed an inadequate first response performance during simulated cardiac arrest with regard to no flow ratio and prolonged defibrillation. Future educational and organizational interventions should focus on improving the quality of care during the early phase of resuscitation with regards to continuing chest compressions and early defibrillation as well as evaluating the educational interventions' impact on clinical performance and patient outcome.

## List of abbreviations

CPR: cardiopulmonary resuscitation; ERC: European Resuscitation Council; NFT: no flow time; NFR: no flow ratio: ROSC: return of spontaneous circulation.

## Competing interests

There are no financial or non-financial competing interests for any of the authors. The study was financed by the Karola Jørgensen's Research Foundation (Karola Jørgensens Forskningsfond). The sponsor had no role in the design and conduct of the study, the interpretation of data, or in preparation and approval of the manuscript.

## Authors' contributions

FM contributed to the conception and design of the study, the funding, the acquisition, analysis and interpretation of data, and contributed to the drafting the manuscript. MB and LF contributed to the study conception and design, the acquisition, analysis and interpretation of data. JO and KRW contributed in the acquisition of data as well as interpretation of data. NPS and TK contributed to the study conception and design, analysis and interpretation of data. All authors contributed to the revision of the manuscript and approved the final article for publication.
